# Supplementary material for: Tumor Microenvironment Responsive Nanomicelle with Folic Acid Modification Co-Delivery of Doxorubicin/Shikonin for Triple Negative Breast Cancer Treatment
Source: Pharmaceuticals (Basel). 2023 Mar 1;16(3):374. doi: 10.3390/ph16030374 (PMC10055947; doi:10.3390/ph16030374)
Supplement: Supplementary file 1 [file pharmaceuticals-16-00374-s001.zip › pharmaceuticals-2157435-supplementary.pdf]

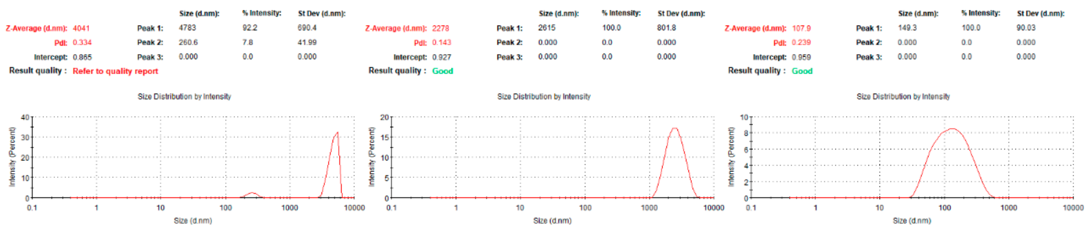

Figure S1. Hydrodynamic dimensions of FPD NMs (FA-PEG-COOH : DOX = 10 : 3, 10 : 2 and 10 : 1 from left to right)

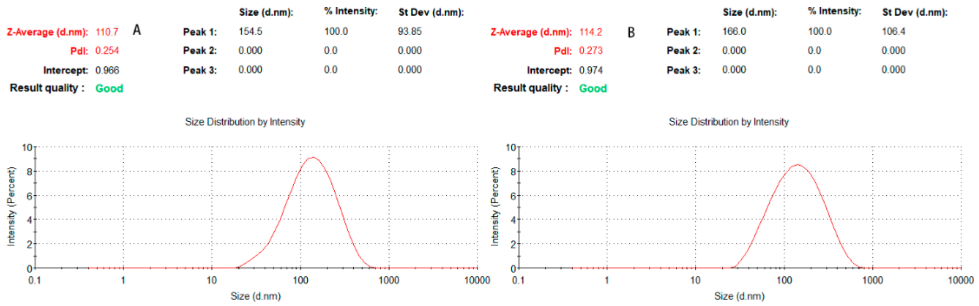

Figure S2. Stability of FPD NM. The hydrodynamic dimension after stewing 48 hours (A) and 72 hours (B) at 4℃.

Table S1. Infrared peak assignment of synthetic materials

Materials	Peak	Origins
FA-PEG-COOH	1718 cm^{-1}	Free carbonyl stretching vibration peak from FA and NH_2-PEG-COOH
	1685 cm^{-1}	Newly amide bond after reaction
	1105 cm^{-1}	Ether bond, C–O–C stretching vibration peak from NH_2-PEG-COOH
	2800-3000 cm^{-1}	Methylene peak from NH_2-PEG-COOH
FA-PEG-DOX	1281 cm^{-1}	vibration C-O peak of the phenol from DOX
	1728 cm^{-1}	Carbonyl stretching vibration peak from DOX
	1681 cm^{-1}	Amide bond merge peak
	2800-3000 cm^{-1}	Methylene peak from NH_2-PEG-COOH

Table S2. ^{1}H NMR peak assignment of synthetic materials

Materials	Peak	Origins
FA-PEG-COOH	3.50 ppm	Methylene hydrogen peak from NH_2-PEG-COOH
	6.5 - 9.0 ppm	Heterocyclic hydrogen peak of FA
FA-PEG-DOX	5.82 pmm, 5.34 pmm, 5.05 pmm and 4.12 pmm	unreacted hydroxyl hydrogen or hydrogen on hydroxyl o-carbon from DOX
	3.50 ppm	Methylene hydrogen peak from NH_2-PEG-COOH
	6.5 - 9.0 ppm	Heterocyclic hydrogen peak from FA and newly benzene peak from DOX
